# Editorial: Decoding the spectrum of plasma cell heterogeneity: insights into maturity and longevity

**DOI:** 10.3389/fimmu.2026.1916518

**Published:** 2026-07-09

**Authors:** Doan C. Nguyen, Julie Tellier, David R. Fooksman, David Allman

**Affiliations:** 1 Department of Medicine, School of Medicine, Emory University, Atlanta, GA, United States; 2 The Walter and Eliza Hall Institute of Medical Research, Parkville, VIC, Australia; 3 Departments of Pathology, Microbiology, and Immunology, Albert Einstein College of Medicine, Bronx, NY, United States; 4 Department of Pathology and Laboratory Medicine, Perelman School of Medicine, University of Pennsylvania, Philadelphia, PA, United States

**Keywords:** antibody, plasma cell, heterogeneity, longevity, maturity

## Introduction

Plasma cells (PC), also referred to as antibody-secreting cells (ASC), are specialized cells in the immune system, capable of producing thousands of antibody molecules per second (1). For much of immunological history, the PC/ASC pool was treated as functionally homogeneous, i.e., a uniform corps of antibody factories distinguished by isotype and antigen specificity (2). Over the past decade, that view has shifted as accumulating evidence has revealed substantial heterogeneity within the PC/ASC compartment (3–5): it is a considerably heterogeneous continuum, spanning from short-lived (SL) ASC that arise within days of antigen encounter and disappear within weeks, to long-lived PC (LLPC) that predominantly reside in the bone marrow (BM) and maintain serological memory throughout a lifetime.

This Research Topic assembled 10 articles that collectively interrogate PC heterogeneity – from multiple angles: developmental, mechanistic, ecological, tissue-specific, technological, and clinical. Together, they provide a coherent argument: understanding what a PC is requires understanding not only what it produces, but also where it came from and resides, how it is induced and survives, and what broader biological and pathological roles it plays.

## From birth to BM: the developmental arc

A foundational question underlying this Research Topic is how a newly minted, SL ASC in peripheral blood becomes a LLPC in the BM (3, 6, 7). Two articles address this trajectory directly and a third provides critical ecological context.

Koike and Ise offer a conceptual map of the journey, highlighting the anatomical segregation between induction sites, i.e., secondary lymphoid organs (SLO), where ASC are first generated, and effector sites, where LLPC reside. The review summaries recent advances in identifying LLPC precursors among newly generated ASC in SLO and traces the molecular programs governing their subsequent migration and maturation within the BM. Both cell-intrinsic programs and extrinsic niche signals emerge as indispensable: longevity is not an intrinsic property present at ASC birth, but progressively acquired as the cells adapt into supportive microenvironments.

That maturation process has a measurable functional correlate. Nguyen et al. report experimental evidence antibody secretion is not static across the ASC lifespan. Using bulk and single-cell cultures in an *in vitro* BM mimetic survival system, the authors show mature BM ASC produce more IgG per cell than early-minted blood counterparts. Critically, blood ASC cultured in supportive conditions increase secretion rates overtime and acquire LLPC-like phenotypes, establishing functional output is coupled to maturational stage. This work provides a quantitative dimension to the developmental arc described by Koike and Ise, anchoring the PC biology in humans.

How much room exists in the BM for this maturation to occur at scale? Tonouchi et al. tackle this question with an elegant pulse-labeling strategy in mice. Their findings demonstrate active gut-associated germinal centers (GC) continuously seed the BM with new PC, with GC-derived PC accumulating to represent ~1/3 BM PC examined at 70–80 days. The findings suggest the BM PC compartment remains dynamic with continuous inputs from ongoing immune responses, appertaining to vaccine durability and competitive niche occupancy.

## Heterogeneity as principle, not exception

If the developmental arc explains the temporal dimension of PC diversity, the heterogeneity axis captures its depth. Multiple articles examine why PC with similar origins can behave differently (beyond age and location).

Reinke et al. provide a molecular account of LLPC survival, focusing on the receptor-signaling networks, particularly TACI and CD28, that support LLPC to engage survival signals and upregulate anti-apoptotic machinery. The review highlights how LLPC differ from SL ASC: by a constellation of cell-intrinsic programs unlocked by the BM microenvironment. While the question of why some PC access this longevity program and others do not remains incompletely answered, this review sharpens the molecular lexicon for pursuing it.

Akessé and Bonaud broaden the conceptual frame by cataloguing the multiple axes along which PC diversity is shaped. B cell precursor identity, antigen affinity, BCR signaling strength, Ig isotype, microenvironments, cytokines, cellular interactions, and temporal variables – including age, sex, and inflammatory status – all contribute to PC fate. The authors introduce the concept of “Moment” to capture how these temporal and contextual variables modulate PC differentiation throughout maturation, arguing for a model in which heterogeneity is an adaptive feature of the humoral responses. This framework complements the mechanistic detail in Reinke et al. by offering a higher-order view of the landscape.

The methodological foundations for studying PC heterogeneity are addressed by Pioli and Pioli, who survey the contribution of genetically modified mouse models to the field. From early transgenic reporters marking PC identity to modern timestamping approaches allowing tracking of PC origins and lineage trajectories, the review maps the technical infrastructure for biological discoveries. This perspective piece underscores advances in understanding PC heterogeneity have been coupled to the availability of refined mouse models and points forward to where next-generation tools may take the field.

## Ecology and the tissue dimension

PC do not exist in isolation: their survival, identity, and functions are shaped by the tissues they reside (8). Multiple contributions examine this spatial dimension.

The niche perspective offered by Tonouchi et al. is complemented by two reviews that expand its geographical frame. Wang et al. summarize the biology governing the circulating PC (CPC) pool, a small but diagnostically significant fraction of PC persistent in peripheral blood. Beyond a role as proxies for immune activation, CPC are emerging as biomarkers across infections, autoimmune diseases, and PC malignancies, with recent guidelines recognizing elevated CPC as an adverse prognostic feature in multiple myeloma. This article bridges PC biology to liquid biopsy approaches that reshape disease monitoring.

Merder and Moseman extend the tissue map further. The authors examine PC that accumulate within the central nervous system (CNS) and its border zones (meninges, olfactory mucosa, and brain parenchyma). Traditionally viewed as sites poorly accessible to humoral immunity, the CNS and its borders are increasingly recognized as locations where PC function in both defense and pathology. The review recaps how local PC differentiation, migration, and persistence in the CNS contribute to neuroinvasive infection responses, CNS-targeting autoimmunity, and tumor immunology. It suggests the parallels with LLPC biology in the BM: specialized niches support local PC persistence across multiple tissues, inquiring whether general principles of niche-driven longevity operate throughout the body.

## Translational convergence

The translational implications of this field run in two directions: exploiting PC biology for vaccine design and understanding its dysregulation in disease.

Cusimano et al. address the vaccine design direction with imperativeness. Despite LLPC being the ultimate target of most immunization strategies, generation of vaccine-induced LLPC (i.e., long-lived vaccines (LLV)) remains poorly understood (and inconsistently achieved). The authors summarize emerging technologies, including ELISpot refinements, Ig Trap systems, Nanovials, and single-cell RNA sequencing, that begin to close the measurement gap between LLPC induction and durable antibody titers. The review connects with PC biology synopsized by Koike and Ise, Nguyen et al*.*, and Reinke et al*.*, translating mechanistic insights into a design roadmap for next-generation LLV.

The pathological pole of PC biology is represented by Xu et al., which examines PC mastitis (PCM), a chronic inflammatory breast disorder characterized by prominent PC infiltration in mammary tissue. Though anatomically distinct from the BM and immunologically distinct from myeloma, PCM offers a window into what dysregulated tissue-resident PC activity looks like *in vivo*. This recap of autoimmune, microbial, and microenvironmental etiological factors resonates with broader themes of niche-dependent PC behavior explored elsewhere in this Research Topic *(*Koike and Ise, Reinke et al*.*, Merder and Moseman*).*

## A unified picture

Together, these 10 articles sketch a unified picture of PC biology where heterogeneity serves as the organizing principle. Development is not a single track but a branching program shaped by precursor identity, affinity selection, and niche access. Survival is not intrinsic but achieved through engagement with supportive microenvironments. Function is not static but amplified as cells mature. Location is not irrelevant but constitutive: from BM to meninges and from peripheral blood to the mammary gland, the tissue context shapes what a PC is and behaves.

These articles also converge on a practical message: the availability of the tools to decode PC heterogeneity. Interrogation of PC diversity has been enabled by single-cell sequencing, genetic timestamping, *in vitro* mimetic systems, and novel detection technologies, which were essentially inaccessible a decade ago. What remains is to further integrate these approaches across human and mouse systems, at the interface of basic and translational science, and across the health-disease continuum.

The editors thank all contributing authors and reviewers for commitment to this Research Topic. While the work assembled here does not demystify PC heterogeneity, it advances the lexicon and the conceptual frameworks to pursue it.
